# Repeated Blockade of NMDA Receptors During Adolescence Impairs Reversal Learning and Disrupts GABAergic Interneurons in Rat Medial Prefrontal Cortex

**DOI:** 10.3389/fnmol.2016.00017

**Published:** 2016-03-03

**Authors:** Ji-Tao Li, Yun-Ai Su, Hong-Li Wang, Ying-Ying Zhao, Xue-Mei Liao, Xiao-Dong Wang, Tian-Mei Si

**Affiliations:** ^1^National Clinical Research Center for Mental Disorders, (Peking University Sixth Hospital/Institute of Mental Health) and the Key Laboratory of Mental Health, Ministry of Health (Peking University)Beijing, China; ^2^Depression Treatment Center, Beijing Anding Hospital of Capital Medical UniversityBeijing, China; ^3^Department of Neurobiology, Key Laboratory of Medical Neurobiology of Ministry of Health of China, Zhejiang Province Key Laboratory of Neurobiology, Zhejiang University School of MedicineHangzhou, China

**Keywords:** adolescence, interneurons, MK-801, prefrontal cortex, rat, reversal learning

## Abstract

Adolescence is of particular significance to schizophrenia, since psychosis onset typically occurs in this critical period. Based on the N-methyl-D-aspartate (NMDA) receptor hypofunction hypothesis of schizophrenia, in this study, we investigated whether and how repeated NMDA receptor blockade during adolescence would affect GABAergic interneurons in rat medial prefrontal cortex (mPFC) and mPFC-mediated cognitive functions. Specifically, adolescent rats were subjected to intraperitoneal administration of MK-801 (0.1, 0.2, 0.4 mg/kg), a non-competitive NMDA receptor antagonist, for 14 days and then tested for reference memory and reversal learning in the water maze. The density of parvabumin (PV)-, calbindin (CB)- and calretinin (CR)-positive neurons in mPFC was analyzed at either 24 h or 7 days after drug cessation. We found that MK-801 treatment delayed reversal learning in the water maze without affecting initial acquisition. Strikingly, MK-801 treatment also significantly reduced the density of PV^+^ and CB^+^ neurons, and this effect persisted for 7 days after drug cessation at the dose of 0.2 mg/kg. We further demonstrated that the reduction in PV^+^ and CB^+^ neuron densities was ascribed to a downregulation of the expression levels of PV and CB, but not to neuronal death. These results parallel the behavioral and neuropathological changes of schizophrenia and provide evidence that adolescent NMDA receptors antagonism offers a useful tool for unraveling the etiology of the disease.

## Introduction

It is now widely accepted that schizophrenia is a neurodevelopmental disorder (Fatemi and Folsom, 2009). In the past decades, vast research efforts have been made to identify the genetic and early developmental factors that could contribute to the pathophysiology of the disease. Given that psychosis onset typically occurs during adolescence, this critical developmental period is of obvious significance for schizophrenia. However, until recently, few studies have attempted to investigate what maturational processes during adolescence are involved in the etiology of schizophrenia (Rujescu et al., 2006; Braun et al., 2007; Thomsen et al., 2010; Li et al., 2011; Le Magueresse and Monyer, 2013; Thomases et al., 2013) and our understanding of this issue is still limited.

Deficits in the inhibitory gamma-aminobutyric-acid (GABA)-ergic interneurons have been consistently found in the prefrontal cortex (PFC) of schizophrenic patients (Reynolds et al., 2004). These deficits are thought to contribute to the disturbances of PFC-dependent cognitive functions in schizophrenia, since GABAergic transmission is critical for normal cognitive functions via producing synchronized network oscillations (Uhlhaas et al., 2006; Gonzalez-Burgos et al., 2011). Notably, the functional maturation of the GABAergic interneurons lasts until late adolescence and it has been hypothesized that schizophrenia may be associated with the disrupted development of these interneurons (Di Cristo, 2007; Hoftman and Lewis, 2011; Caballero et al., 2014; Gonzalez-Burgos et al., 2015). Of particular interest, the maturation of GABAergic interneurons involves the N-methyl-D-aspartate (NMDA) receptors (Thomases et al., 2013), which are highly relevant to the pathophysiology of schizophrenia, considering that blocking the NMDA receptors using its antagonists, such as phencyclidine (PCP), MK-801 or ketamine, constitutes one of the most widely used animal models for schizophrenia (Jentsch and Roth, 1999). Accordingly, investigating how repeated blockade of NMDA receptors during adolescence would affect GABAergic interneurons in the PFC would provide insight to the mechanisms underlying the onset of the disease.

Three nonoverlapping classes of cortical GABAergic interneurons can be identified according to the calcium-binding proteins (CBPs) they contain: parvalbumin (PV), calbindin (CB) and calretinin (CR), which together account for more than 80% of total interneurons in rat medial prefrontal cortex (mPFC; Gabbott et al., 1997). To our knowledge, few studies have examined the effects of repeated NMDA receptor antagonism during adolescence on various types of interneurons in the mPFC (Braun et al., 2007; Thomsen et al., 2010). Among these studies, conflicting results were reported: whereas Braun et al. (2007) reported no significant change of PV-positive (PV^+^) and CR^+^ cell counts in animals receiving MK-801, Thomsen et al. (2010) noted reduced PV mRNA expression by PCP treatment. Moreover, these effects were examined shortly after drug cessation and it remains unknown about the long-term influence of such treatment.

In this study, we aimed to investigate whether and how subchronic NMDA receptor blockade during adolescence would affect PFC-dependent cognitive functions and the density of three subtypes of GABAergic interneurons in this region. Previously, we have reported that subchronic MK-801 treatment regimen induced deficits in the spatial working memory that is mediated by mPFC (Li et al., 2011). Here, reversal learning in the Morris water maze was conducted to further validate the impairment of mPFC-dependent functions. We then assessed the effects of MK-801 treatment on the three CBP-defined subtypes of interneurons by cell counting at 24 h or 7 days after drug cessation. Because the reduced cell densities could reflect either a downregulation of CBP expression levels or a loss of neurons, we further measured the number of apoptotic cells and the expression levels of apoptosis-related molecules (Bcl-2 and Bax) and three CBPs in the mPFC to reveal which mechanism is responsible for the deficits in GABAergic interneurons induced by repeated NMDA receptor blockade during adolescence.

## Materials and Methods

### Animals and Drug Treatment

Adolescent male Sprague-Dawley (SD) rats (*n* = 92, age 28 days, weighing 90 ± 5 g) were used in the present study. Purchased from the Laboratory of Animal Science (Peking University Health Science Center, Beijing, China) after weaning at postnatal day (PND) 21, animals were housed four per cage in a controlled environment (23 ± 1°C; 45–55% relative humidity; fixed 12/12 h light/dark cycle, lights on at 08:00 h) with food and water *ad libitum*. All procedures were performed in accordance with the National Institute of Health’s Guide for the Use and Care of Laboratory Animals and were approved by the Peking University Committee on Animal Care and Use.

**Figure 1:**
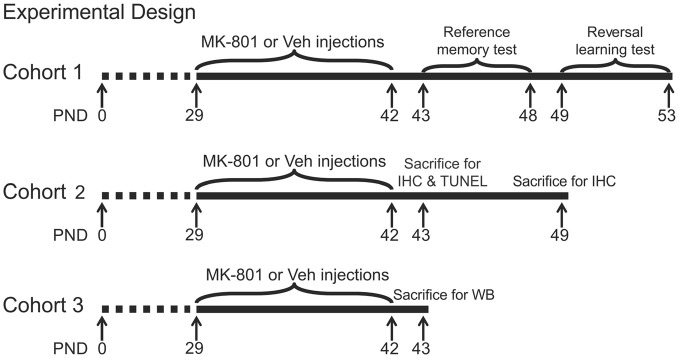
**Experimental timelines of behavioral and neurochemical procedures in three animal cohorts.** PND, postnatal day; IHC, immunohistochemistry; TUNEL, terminal deoxynucleotidyl transferase-mediated UTP nick end labeling; WB, western blot.

MK-801 (dizocilpine, St. Louis, MO, USA) was dissolved in saline and stored at 4°C. After 7-day habituation in the animal facility, animals started to receive drug administration on PND 29. They were randomly assigned into four groups (*n* = 23 per group) and were given daily intraperitoneal injection (2 ml/kg body weight) of MK-801 at doses of 0.1, 0.2, 0.4 mg/kg, or 0.9% saline (vehicle) between 09:00 and 10:20 h for 14 days. Body weights of animals were recorded daily during drug treatment. Four animals from each group were sacrificed at 24 h or 7 days after the last MK-801 treatment for immunohistochemistry. Another five animals per group were sacrificed 24 h after last MK-801 treatment for Western blot. The other animals were tested in the Morris water maze for reference memory 24 h after drug cessation and reversal learning 7 days after drug cessation. A summary of animal cohorts and the timing of various procedures are shown in Figure 1.

### Morris Water Maze Testing

The Morris water maze was used to assess reference memory and reversal learning according to protocols previously reported (Vorhees and Williams, 2006). A circular pool (185 cm in diameter, 45 cm in height, made from dark plastic) was filled with tap water (thermostatically controlled at 22 ± 1°C). A circular platform (9 cm in diameter) was submerged 1 cm below the water surface for rats to climb onto once its presence was detected. The pool was surrounded by a white curtain, to which several pieces of colored paper in different geometric shapes were attached to help rats obtain spatial orientation. A camera suspended above the pool center was used for recording the swimming path of the rats and video outputs were analyzed using a video tracking and analysis system.

The reference memory task consisted of five consecutive days of training to acquire the location of the hidden platform, followed by a probe test on the sixth day without the platform. The pool was divided into four equal imaginary Quadrants (I, II, III and VI) and the platform was located at the center of Quadrant III. On each training day, rats received four swimming trials with each starting from different locations. Animals finding the platform within 60 s were allowed to sit on it for 15 s and those failing to do so were guided by the experimenter to the platform and allowed to sit on it for 15 s. The inter-trial interval was 20 s. After training, animals were dried with a towel and put in a clean cage to avoid interaction with other animals. In the probe test, rats were placed in the pool without the platform to swim for 60 s and their swimming time in each quadrant was collected and analyzed.

The reversal learning task was performed after the reference memory task. The platform was moved to the center of the opposite quadrant (from Quadrant III to Quadrant I). Animals were trained for four consecutive days followed by a probe trial on the fifth day. The procedure was performed as described above for the reference memory task.

Water maze performance in the acquisition phase was expressed as the mean escape latency to the platform of four trials in a training day. For the probe tests, the percentage time animals swam in the target, adjacent and opposite quadrants were used as an indication of spatial memory. The data of one animal in the group of MK-801 0.1 mg/kg and one in the group of MK-801 0.4 mg/kg were excluded from analysis, because they frequently jumped off the platform.

### Immunohistochemistry

Immunohistochemistry was performed to quantify the number and density of all GABAergic cells (identified as the cells showing positive staining of the GABA synthesizing enzyme, glutamic acid decarboxylase (GAD), which in adult brain has two major isoforms, GAD65 and GAD67) and three major subtypes of GABAergic interneurons (PV^+^, CB^+^ and CR^+^ cells). At each sacrifice time point, animals (*n* = 4 per group) under pentobarbital anesthesia were transcardially perfused with 250 ml cold saline solution, followed by 250 ml of 4% paraformaldehyde in 0.1 M phosphate-buffered saline (PBS, pH 7.4). Brains were removed, post-fixed in the same fixative overnight and stored in 10, 20 and 30% sucrose solution at 4°C for dehydration. The brains were then quenched in cold N-hexane at −60°C for 20 s and stored at −80°C until required.

Serial coronal sections of the PFC (30 μm thick, bregma 3.72–2.52; Paxinos and Watson, 2005) were collected on a cryostat (Leica, Wetzlar, Germany). Free-floating sections were washed three times for 5 min in 0.1 M PBS and incubated in 3% hydrogen peroxide for 10 min, followed by washing steps and 90 min incubation in 1% normal goat serum. They were labeled with the following primary antibodies overnight at 4°C: GAD65/67 (rabbit anti-GAD65/67, 1:500; ab11070, Abcam, Cambridge, UK), PV (rabbit anti-Parvalbumin, 1:20,000, PV 25, Swant, Switzerland), CB (rabbit anti-calbindin, 1:20,000, CB-38a, Swant), and CR (rabbit anti-calretinin, 1:5000, 7699/3H, Swant). The next day, all slices were rinsed, incubated with secondary antibodies (goat anti-rabbit IgG(H+L)/Biotin and goat anti-mice IgG(H+L)/Biotin, Zhongshan Gold Bridge Biotechnology, China) for 90 min at room temperature. After rinsing, the 3,3′-Diaminobenzidine Horseradish Peroxidase Color Development Kit was used for 5 min slice staining. Finally the sections were transferred onto slides and coverslipped.

**Figure 2:**
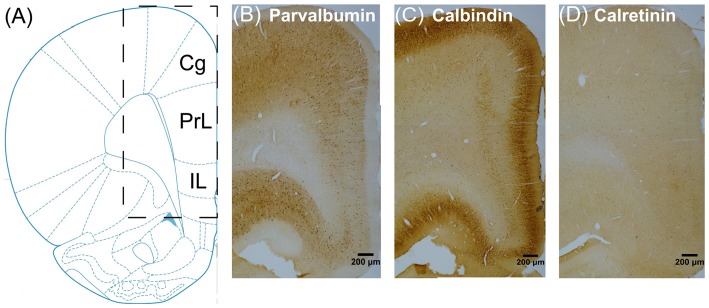
**Schematic of three subregions of rat medial prefrontal cortex (mPFC), including the cingulate cortex (Cg), the prelimbic cortex (PrL) and infralimbic cortex (IL), for immunohistochemistry analyses (A) and representative sections in the vehicle group immunostained for PV^+^ (B) CB^+^ (C) and CR^+^ (D) neurons**.

Images were obtained at 100× using an Olympus BX51 microscope equipped with a charge-coupled device (CCD) camera (CoolSNAP MP5, Roper Scientific Corporation, USA) and analyzed by the NIH ImageJ Software (National Institutes of Health, Bethesda, MD, USA). We focused on three subregions of mPFC (Figure 2): the cingulate cortex (Cg), the prelimbic cortex (PrL) and infralimbic cortex (IL; Paxinos and Watson, 2005). To count the number of inhibitory interneurons within each subregion, we defined regions of interest (ROI) as three nonoverlapping identical rectangles distributed evenly within each subregion covering from the pial surface to the white matter border (rectangle size: Cg 800 μm × 100 μm, PrL 600 μm × 100 μm, IL 500 μm × 100 μm). The positive stained PV^+^, CB^+^, CR^+^ and GAD65/67^+^ cells were counted within each ROI, which were then averaged and divided by the area of the rectangle to obtain the relative cell densities.

### Double-Fluorescence Immunohistochemistry

Since a subpopulation of pyramidal neurons in both superficial and deep layers of mPFC was weakly stained for CB, as shown in our own results and reported in previous studies (Gabbott et al., 1997; Gonchar and Burkhalter, 1997; Caballero et al., 2014), it is difficult to differentiate bonafide interneurons from pyramidal neurons. To label specific CB^+^ interneuron populations, we performed double-labeling immunofluorescence to identify neurons that co-express CB and somatostatin (SST), another selective interneuron marker. This procedure was carried out on free-floating sections as described previously (Chen et al., 2004). Briefly, after incubation with primary antibodies (CB, rabbit anti-calbindin, 1:5000, CB-38a, Swant; SST, goat anti-somatostatin, 1:100, sc-7819, Santa Cruz Biotechnology) overnight at 4°C, sections were rinsed and labeled with anti-rabbit Alexa Fluor 488- and anti-goat Alxea Fluor 594-conjugated donkey secondary antibodies (1:500, Invitrogen, Karlsruhe, Germany) for 2 h at room temperature. After rinsing, sections were transferred onto slides, dried and coverslipped with Vectashield mounting medium containing 4′,6-diamidino-2-phenylindole (Vector laboratories, Burlingame, CA, USA).

The fluorescent images (1024 × 1024 pixels) were obtained with an Olympus IX81 confocal microscope (Olympus, Tokyo, Japan) at 10× magnification using the Kalman filter and sequential scanning mode under identical settings for laser power, photomultiplier gain and offset. For the colocalization assessment of CB and SST, images were adjusted for better brightness and contrast using the FV10-ASW 1.7 Software (Olympus). The number of CB^+^ neurons and the number of interneurons that co-express CB and SST were then quantified using the NIH ImageJ Software.

### Terminal Deoxynucleotidyl Transferase-Mediated UTP Nick End Labeling (TUNEL) Assay

To measure apoptotic cell death, TUNEL assay was conducted according to the protocol of the manufacturer of the *in situ* cell death detection kit (Roche). Briefly, after incubation with the blocking solution (3% H_2_O_2_ in methanol) for 10 min at room temperature, free-floating brain sections were rinsed, incubated with the permeabilization solution (0.1% Triton X-100 in 0.1% sodium citrate) for 2 min at 4°C and labeled with the TUNEL reaction mixture for 60 min at 37°C. After rinsing, sections were then transferred onto slides, dried and coverslipped. TUNEL staining images were obtained using an Olympus IX71 microscope and TUNEL^+^ neurons were counted using the NIH ImageJ Software.

### Western Blot

Using Western blot, we examined the protein levels of GAD65/67, two apoptosis-related molecules (Bcl-2 and Bax) and three CBPs (PV, CB and CR). Twenty-four hours after last drug treatment, rats were deeply anesthetized with pentobarbital and their brains rapidly removed and dissected to obtain mPFC as previously described (Gearhart et al., 2006). Tissue from individual rats was immediately homogenized on ice in ice-cold lysis buffer [137 mM NaCl, 20 mM Tris–HCl (pH 8.0), 1% NP-40, 10% glycerol, 1 mM PMSF, 10 mg/ml aprotinin, 1 mg/ml leupeptin, 0.5 mM sodium vanadate], sonicated, and centrifuged. The supernatants were stored at −80°C until required.

Samples containing 20 μg of protein were resolved by 10% (for GAD65/67, CB and CR), 12.5% (for Bcl-2 and Bax) acrylamide gels using Laemmli–SDS-PAGE, or 15% (for PV) acrylamide gels using Tricine-SDS-PAGE (Schagger, 2006), and transferred electrophoretically to a polyvinylidene difluoride (PVDF) membrane (Millipore, Bedford, MA, USA). The PVDF membranes with the pore size being 0.2 μm for PV and 0.45 μm for other proteins containing the proteins of interest were then blocked with 5% non-fat milk diluted in Tris-buffered saline-Tween (TBST) [150 mM NaCl, 10 mM Tris-HCl (pH 7.5) and 0.1% Tween] for 1 h at room temperature and incubated overnight at 4°C in primary antibodies diluted in TBST containing 5% non-fat milk (GAD65/67: rabbit anti-GAD65/67, 1:5000, ab11070, Abcam; Bcl-2: rabbit anti-Bcl-2, 1:1000, ab7973, Abcam; Bax: rabbit anti-Bax, 1:1000, ab32503, Abcam; PV: rabbit anti-Parvalbumin, 1:2000, 195002, Synaptic Systems, Germany; CB: rabbit anti-calbindin, 1:20,000, CB-38a, Swant; CR: rabbit anti-calretinin, 1:10,000, 7699/3H, Swant; β-actin: mouse anti-β-actin, 1:20,000, 3700, Cell Signaling, Danvers, MA, USA). The next day, membranes were rinsed three times with TBST (8 min each time) and incubated for 2 h with horseradish peroxidase-conjugated goat anti-rabbit or anti-mouse secondary antibodies (1:2500–20,000, Santa Cruz Biotechnology) diluted in TBST containing 5% non-fat milk. Following another three TBST rinses, proteins of interest were visualized using an ECL system (Pierce, Rockford, IL, USA) and Kodak XBT-1 film. The immunoreactive signals of the target proteins were quantified by densitometry and the values were corrected based on their corresponding β-actin levels. All results were normalized by taking the value of the vehicle group as 100%.

### Statistical Analyses

All the results were expressed as the mean ± SEM. The learning performance during the training period was analyzed using repeated measures analysis of variance (ANOVA) with training day as a within-subject factor and group as a between-subject factor. The percentage time animals swam in the target, adjacent and opposite quadrants in the probe tests was compared with each other using Wilcoxon signed rank test. The number of PV^+^, CB^+^, CR^+^, GAD65/67^+^ and TUNEL^+^ neurons in three mPFC subregions was analyzed using two-way ANOVA with group and subregion (Cg, PrL and IL) as the factors and the *post hoc* analyses of main effects of group were conducted using the Fisher’s least significant difference (LSD) test. One-way ANOVA, followed by the Fisher’s LSD test was used to examine treatment effects in various measures, including the escape latency within each training day, the time spent in the target quadrant during probe tests, the cell counts within each mPFC subregion and the western blot results. The significance level for all statistical tests was *P* < 0.05.

## Results

### Subchronic MK-801 Treatment During Adolescence Delayed Reversal Learning in the Water Maze

**Figure 3:**
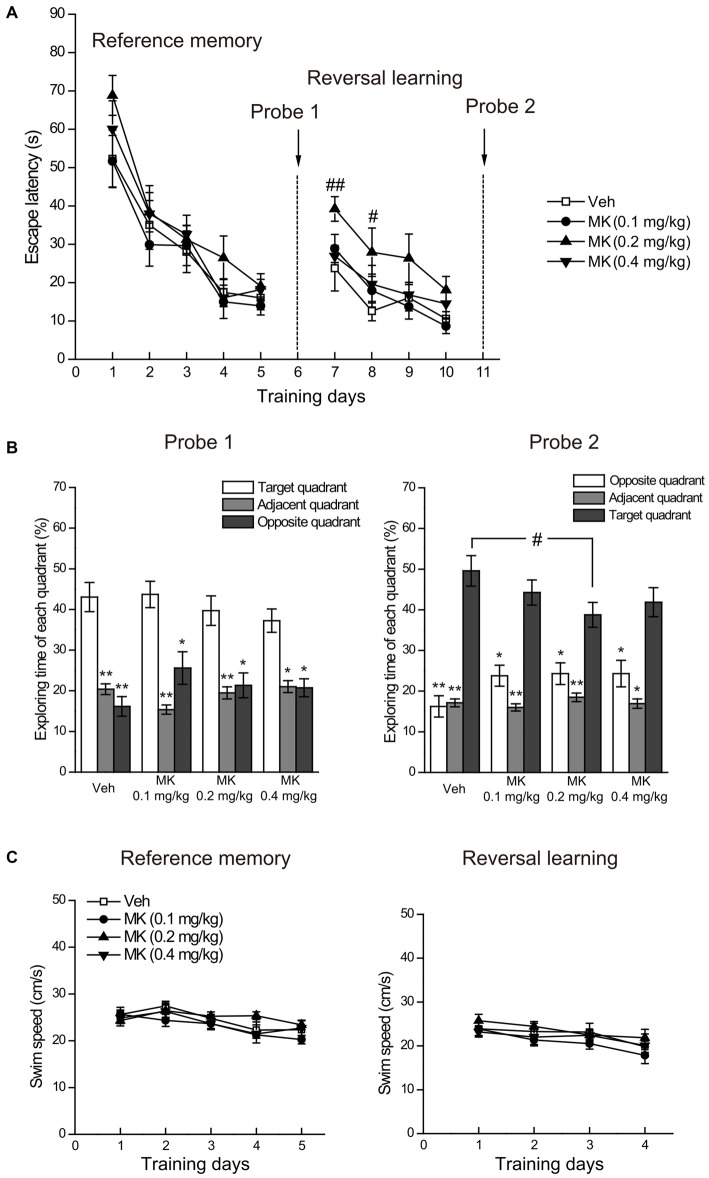
**Effects of subchronic treatment of MK-801 (0.1, 0.2, 0.4 mg/kg, once daily) during adolescence (PND 29–42) on reference memory and reversal learning in the Morris water maze. (A)** Mean escape latency in the reference memory and reversal learning tasks over training days. **(B)** The percentage time animals swam in the target, adjacent and opposite quadrants during the probe tests. **(C)** Mean swim speed in the reference memory and reversal learning tasks over training days. *n* = 9–10 per group. *p < 0.05, **p < 0.01 compared with the target quadrant. ^#^*p* < 0.05 compared with the vehicle group.

We found that subchronic MK-801 treatment during adolescence did not affect reference memory in the Morris water maze (Figure 3A). Specifically, the escape latency to the platform decreased over the 5 days of training (main time effect: *F_(4,136)_* = 67.21, *P* < 0.001), indicating that animals spent less time reaching the platform as the training proceeded. There was a lack of main group effect (*F_(3,34)_* = 1.12, *P* = 0.36) and group × day interaction (*F_(12,136)_* = 0.65, *P* = 0.79), suggesting that spatial learning performance was comparable among all treatment groups. This result was confirmed by the probe test (Figure 3B, left side panel), in which all the groups exhibited the highest spatial preference for the target quadrant. Direct comparison of the percentage time animals swam in the target, adjacent and opposite quadrants using Wilcoxon signed rank test showed that all animals spent significantly more time swimming in the target than other quadrants (*P*s < 0.05) and no significant treatment effect was found for the time spent in the target quadrant.

Regarding the performance in the reversal learning task (Figure 3A), repeated measures ANOVA on the escape latency to the platform over the 4 days of training showed significant main effects of day (*F_(3,102)_* = 20.83, *P* < 0.001) and group (*F_(3,34)_* = 3.25, *P* = 0.034), but no interaction of the two factors (*F_(9,102)_* = 0.59, *P* = 0.80). Looking at group differences in the training session day by day, we found that compared with the control group, only animals in the group of MK-801 0.2 mg/kg spent more time in relocating the platform location at the acquisition Day 1 (*P* = 0.009) and Day 2 (*P* = 0.024). Similar trends for this group were observed in the following training days, although statistical significance was not reached. These results suggest that animals receiving 0.2 mg/kg of MK-801 had delayed reversal learning compared to the control animals. After training, the probe test (Figure 3B, right side panel) revealed that the four groups successfully differentiated the target from other quadrants (*P*s < 0.05), indicating that all the animals eventually acquired the new platform position. Additionally, we noticed a subtle but statistically significant difference between MK-801 0.2 mg/kg group and the control group concerning the time in the target quadrant (*P* = 0.027), which suggests that 0.2 mg/kg of MK-801 treatment may mildly compromise memory retrieval performance.

Finally, we analyzed swimming speed during the acquisition phases of reference memory and reversal learning to determine whether MK-801 induces general sensorimotor deficits (Vorhees and Williams, 2006). As shown in Figure 3C, there was no significant difference in swimming speed among treatment groups, thus ruling out the possibility that differential performance in reversal learning may be ascribed to differences in general sensorimotor skills.

### Subchronic MK-801 Treatment During Adolescence Decreased PV^+^ and CB^+^ Cell Densities in the mPFC

**Figure 4:**
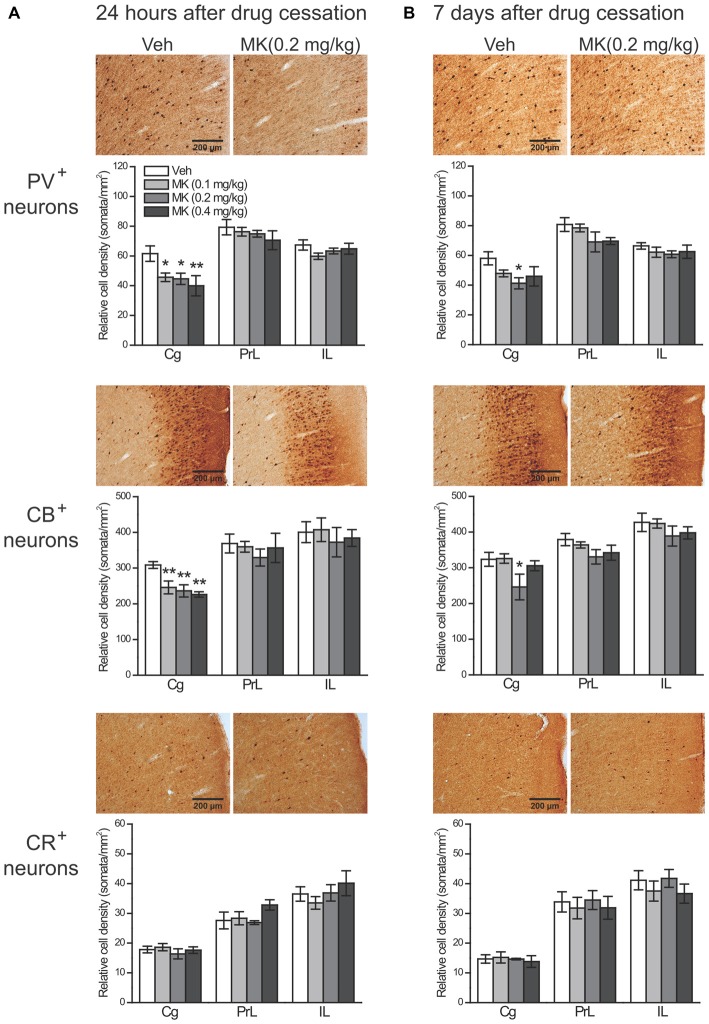
**Effects of subchronic treatment of MK-801 (0.1, 0.2, 0.4 mg/kg, once daily) during adolescence (PND 29–42) on relative density of PV^+^, CB^+^ and CR^+^ neurons in rat mPFC 24 h (A) and 7 days (B) after drug cessation.** Representative sections show immunoreactivity of each type of neurons in the cingulate subregion in animals receiving either vehicle or MK-801 (0.2 mg/kg) treatment. *n* = 4 per group. *p < 0.05, **p < 0.01 compared with the vehicle group. Cg, cingulate cortex; PrL, prelimbic cortex; IL, infralimbic cortex.

Twenty Four hour after drug cessation (Figure 4A), in the cell counts of PV^+^ interneurons, two-way ANOVA revealed significant main effects of group (*F_(3,36)_* = 4.03, *P* = 0.014) and subregion (*F_(2,36)_* = 43.12, *P* < 0.001), but no significant interaction effect of group × subregion was observed (*F_(6,36)_* = 1.20, *P* = 0.33). *Post hoc* analyses of the main effect of group showed that MK-801 treatment at all the doses induced a significant reduction in the cell counts of PV^+^ interneurons (0.1 mg/kg, *P* = 0.014; 0.2 mg/kg, *P* = 0.018; 0.4 mg/kg, *P* = 0.003). The reduction was primarily found in the Cg (0.1 mg/kg, −26%, *P* = 0.04; 0.2 mg/kg, −28%, *P* = 0.03; 0.4 mg/kg, −35%, *P* = 0.009), but not in other subregions.

As for CB^+^ cells, it is notable that a subpopulation of pyramidal neurons in both superficial and deep layers was weakly stained for CB, as reported in previous studies (Gabbott et al., 1997; Gonchar and Burkhalter, 1997; Caballero et al., 2014). Failing to differentiate *bonafide* interneurons from pyramidal neurons, we therefore counted the number of CB^+^ neurons instead of CB^+^ interneurons. Two-way ANOVA only revealed a significant main effect of subregion (*F_(2,36)_* = 29.66, *P* < 0.001), without a main effect of group (*F_(3,36)_* = 1.84, *P* = 0.16) and the group × subregion interaction (*F_(6,36)_* = 0.50, *P* = 0.80). However, directly examining the treatment effect within each subregion showed that the cell counts of CB^+^ neurons in the Cg were also significantly decreased by MK-801 treatment (0.1 mg/kg, −20%, *P* = 0.007; 0.2 mg/kg, −24%, *P* = 0.003; 0.4 mg/kg, −27%, *P* = 0.001), whereas CB^+^ neurons in other subregions were unaffected.

**Figure 5:**
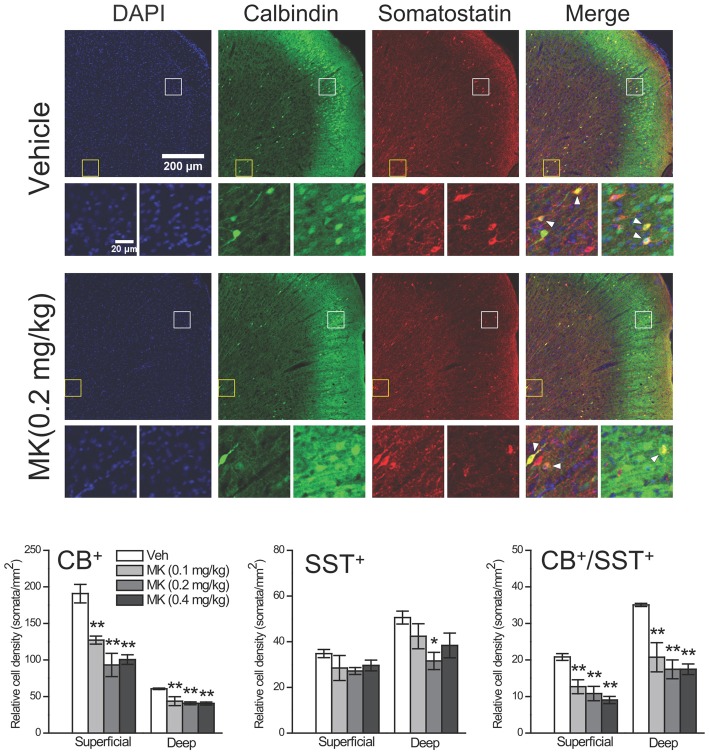
**Effects of subchronic treatment of MK-801 (0.1, 0.2, 0.4 mg/kg, once daily) during adolescence (PND 29–42) on relative density of CB^+^ interneurons that co-express somatostatin (SST) in the superficial and deep layers of the cingulate subregion 24 h after drug cessation.** Representative confocal images show both superficial and deep layers of the cingulate subregion immunostained for DAPI, CB and SST in animals receiving either vehicle or MK-801 (0.2 mg/kg) treatment. Interneurons in superficial (white box) and deep (yellow box) layers are shown in the bottom row. CB^+^/SST^+^ interneurons are labeled with arrowhead. *n* = 4 per group. *p < 0.05, **p < 0.01 compared with the vehicle group.

To further explore the possibility that the CB^+^ neuronal density reduction could occur within specific interneuron populations, we labeled a group of interneurons that co-express CB and SST, another selective interneuron marker, within the Cg subregion using double-fluorescence immunohistochemistry (Figure 5). Considering the layer-specific distribution of CB^+^ neurons, i.e., the majority of weakly CB^+^ neurons were found in superficial layers (Figure 4, middle panel), we analyzed the superficial and deep layers separately. In both types of layers, the cell counts of CB^+^ neurons were significantly reduced in MK-801-treated animals compared to control animals (Superficial layers: 0.1 mg/kg, −33%, *P* = 0.002; 0.2 mg/kg, −51%, *P* < 0.001; 0.4 mg/kg, −47%, *P* < 0.001. Deep layers: 0.1 mg/kg, −27%, *P* = 0.004; 0.2 mg/kg, −32%, *P* = 0.001; 0.4 mg/kg, −33%, *P* = 0.001), which is consistent with our previous immunohistochemical findings. As for the SST^+^ interneurons, while MK-801 treatment did not affect the cell density in the superficial layers, in the deep layers the treatment induced significant reduction in the 0.2 mg/kg group (−38%, *P* = 0.012). For interneurons that express CB in combination with SST, in the vehicle-treated animals, these interneurons accounted for ~11% (21/191) of CB^+^ neurons and ~60% (21/35) of SST^+^ neurons in the superficial layers and the ratios changed to 58% (35/61) and 70% (35/51), respectively, in the deep layers. Importantly, the cell counts of these CB^+^ interneurons were also significantly decreased by MK-801 treatment (Superficial layers: 0.1 mg/kg, −39%, *P* = 0.003; 0.2 mg/kg, −48%, *P* = 0.001; 0.4 mg/kg, −57%, *P* < 0.001. Deep layers: 0.1 mg/kg, −41%, *P* = 0.002; 0.2 mg/kg, −50%, *P* < 0.001; 0.4 mg/kg, −50%, *P* < 0.001).

Seven days after drug cessation (Figure 4B), in the cell counts of PV^+^ interneurons, again significant main effects of group (*F_(3,36)_* = 4.38, *P* = 0.010) and subregion (*F_(2,36)_* = 41.19, *P* < 0.001) were observed without a significant group × subregion interaction (*F_(6,36)_* = 0.56, *P* = 0.76). *Post hoc* analyses revealed that both MK-801 0.2 mg/kg (*P* = 0.002) and 0.4 mg/kg groups (*P* = 0.010) had significantly lower density of PV^+^ neurons in the mPFC, but only the 0.2 mg/kg group exhibited such reduction in the Cg (*P* = 0.022). Similar results were found with CB^+^ neurons, which showed significant main effects of group (*F_(3,36)_* = 4.35, *P* = 0.010) and subregion (*F_(3,36)_* = 27.75, *P* < 0.001), but not group × subregion interaction (*F_(6,36)_* = 0.40, *P* = 0.87). Treatment effects were only observed in the 0.2 mg/kg group, given that the reduction of CB^+^ neurons in this group was significant in the mPFC as a whole (*P* = 0.013) as well as in the Cg (*P* = 0.032).

Notably, at both time points, no significant group effect was observed for CR^+^ interneurons in the mPFC as a whole or in any subregions (Figure 4, bottom panel).

### Repeated NMDA Receptors Blockade Decreased Protein Expression Levels of PV and CB Without Inducing Neuronal Loss

**Figure 6:**
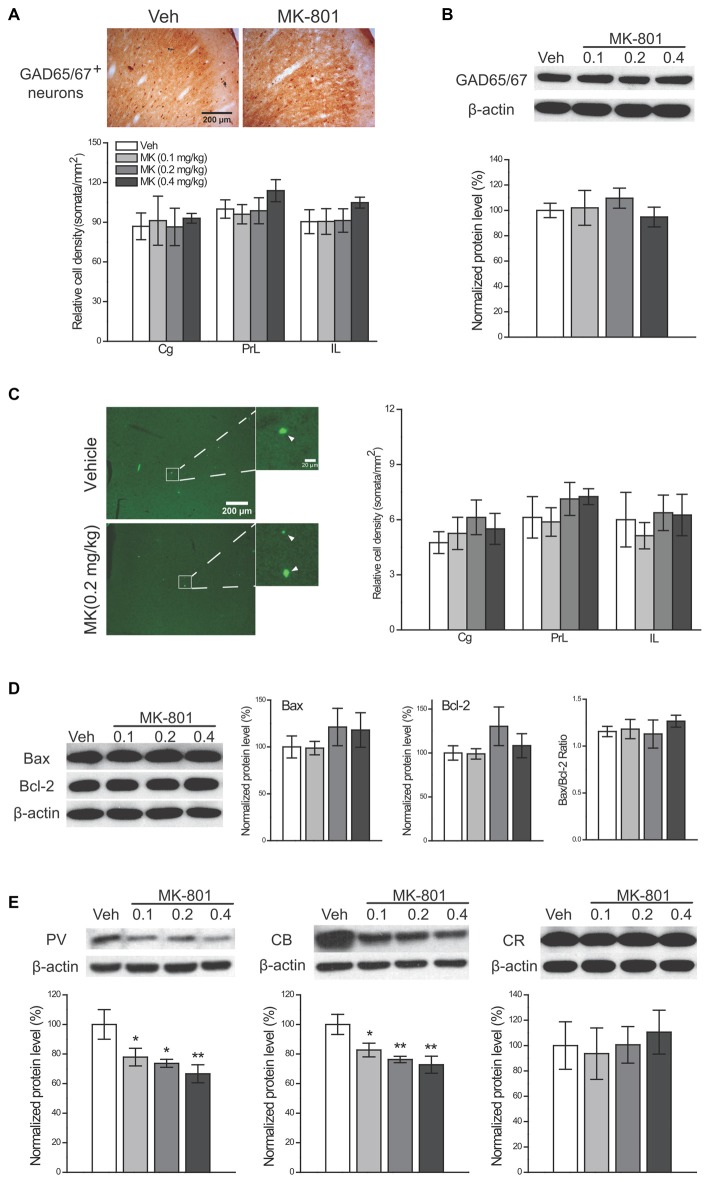
**Investigation of the mechanisms of MK-801-induced reductions in GABAergic interneurons.** Effects of subchronic treatment of MK-801 (0.1, 0.2, 0.4 mg/kg, once daily) during adolescence (PND 29–42) on the density of GAD65/67^+^ neurons **(A)** and the protein levels of GAD65/67 **(B)** the density of apoptotic cells **(C)** the expression levels of two apoptosis-related molecules (Bax and Bcl-2) and their ratio **(D)** and expression levels of three CBPs. **(E)** Representative sections show GAD65/67-immunoreactive neurons **(A)** and apoptotic cells (**C**, arrowhead-labeled), respectively, in the cingulate subregion in animals receiving either vehicle or MK-801 (0.2 mg/kg) treatment. *n* = 4 per group for cell counts and *n* = 5 per group for other results. *p < 0.05, **p < 0.01 compared with the vehicle group. Cg, cingulate cortex; PrL, prelimbic cortex; IL, infralimbic cortex.

To examine whether reduced PV^+^ and CB^+^ neuron densities result from neuronal death or a downregulation of these CBPs, we conducted the following analyses 24 h after drug cessation. First, the number of GAD65/67^+^ interneurons was quantified to evaluate the total number of interneurons. If a real numerical loss of interneurons occurred following MK-801 treatment, we would expect a reduction in the GAD65/67^+^ interneuron density. Nonetheless, no significant change was observed in MK-801-treated animals (Figure 6A), suggesting that reduced densities of PV^+^ and CB^+^ interneurons are unlikely a result of neuronal death. Similarly, the protein levels of GAD65/67 were unaltered by MK-801 treatment (Figure 6B). Moreover, to investigate the potential apoptotic effects of subchronic MK-801 treatment during adolescence, we compared the number of apoptotic cells stained with TUNEL among four treatment groups and did not observe any significant group differences (Figure 6C). The expression levels of two apoptosis-related molecules, Bax and Bcl-2, and their ratio were comparable among four groups as well (Figure 6D), lending further support to the notion that subchronic MK-801 treatment during adolescence had minimal influence on apoptosis. Finally, significant reductions in the expression levels of PV (0.1 mg/kg, −22%, *P* = 0.033; 0.2 mg/kg, −26%, *P* = 0.013; 0.4 mg/kg, −33%, *P* = 0.003) and CB (0.1 mg/kg, −17%, *P* = 0.030; 0.2 mg/kg, −24%, *P* = 0.005; 0.4 mg/kg, −27%, *P* = 0.002), but not CR, in MK-801-treated animals were observed (Figure 6E), indicating that reduced densities of PV^+^ and CB^+^ neurons may be attributed to downregulation of PV and CB expression levels.

## Discussion

In this study, we examined how repeated blockade of NMDA receptors by MK-801 during adolescence would affect PFC-mediated cognitive functions and GABAergic interneurons in rat mPFC. We found that rats receiving MK-801 treatment exhibited delayed reversal learning in the water maze without affecting acquisition. In addition, the treatment reduced the density of PV^+^ and CB^+^ cells in rat mPFC, which persisted for (at least) 7 days after drug cessation in the 0.2 mg/kg group. We also demonstrated that the reduction in PV^+^ and CB^+^ neuron densities resulted from decreased PV and CB expression levels, rather than neuronal death, because at 24 h after drug cessation, the density of apoptotic cells and the expression levels of apoptosis-related molecules (Bcl-2 and Bax) were unaffected by MK-801 treatment, whereas the reduced expression levels of PV and CB in MK-801-treated animals were observed.

Previous studies in adult animals have demonstrated that subchronic treatment of NMDA receptor antagonists including PCP and MK-801 did not affect initial acquisition, but selectively impaired reversal learning (Abdul-Monim et al., 2007; Beninger et al., 2009). Here, for the first time we show that adolescent MK-801 treatment delayed reversal learning in the water maze. The normal performance in the reversal learning task requires cognitive flexibility, which is thought to be mediated by the PFC (Kesner and Churchwell, 2011). Together with our previous finding of the deficits in spatial working memory, another mPFC-dependent cognitive function, by the same treatment regimen (Li et al., 2011), these results suggest that subchronic NMDA receptor blockade during adolescence disrupts mPFC functions.

Cortical interneuron impairments have been hypothesized to contribute to cognitive disturbances in schizophrenia (Lewis et al., 2005). In this study, we found that subchronic MK-801 treatment during adolescence significantly decreased the numbers of PV^+^ and CB^+^ cells in rat mPFC, but did not affect CR^+^ interneurons. This is in line with post-mortem studies of schizophrenia (Daviss and Lewis, 1995; Reynolds et al., 2001; Beasley et al., 2002; Cotter et al., 2002) and animal studies applying chronic NMDA receptor antagonism in neonatal or adult rodents (Cochran et al., 2003; Abekawa et al., 2007; Wang et al., 2008; Turner et al., 2010; Amitai et al., 2012). Our finding is also consistent with a recent study showing that 5 days of PCP treatment in early adolescence resulted in the reduction of the PV mRNA (Thomsen et al., 2010). However, another study in juvenile rats reported that PV^+^ cell counts were unaltered by the low-dose MK-801 treatment from PND 35–56 (Braun et al., 2007). The discrepancy could be explained by dose differences, given that the dose in Braun et al.’s (2007) study (0.02 mg/kg) is much lower than our study (0.1, 0.2 and 0.4 mg/kg). Another possible explanation is that Braun et al.’s (2007) study did not cover the cingulate cortex, the subregion where the most prominent effects were found in our study.

In contrast to the popular research interest in the PV^+^ interneurons, the effects of NMDA receptor blockade on CB^+^ interneurons have been rarely investigated. This class of interneurons deserves more attention actually, given that deficits have been reported in schizophrenic patients (Beasley et al., 2002; Cotter et al., 2002) and related animals models, such as isolation rearing from early adolescence (Harte et al., 2007) and neonatal separation (Helmeke et al., 2008). Here, we found that adolescent MK-801 treatment reduced the numbers of CB^+^ neurons. Given that CB is expressed in a variety of interneurons as well as pyramidal cells, we further demonstrated that the reduction was significant in a population of CB^+^ interneurons that co-express SST, another selective GABAergic neuron marker, suggesting that the cell densities of this specific CB^+^ interneuron population were indeed decreased by adolescent MK-801 treatment.

The reduction in PV^+^ and CB^+^ cell counts may represent a loss of these neurons or a downregulation of PV and CB proteins. Our results about the density of apoptotic cells and expression levels of apoptosis-related molecules and these CBPs support that the latter mechanism is more likely in our study. This is consistent with previous observations that the apoptotic effect of MK-801 is rather limited in adolescent rat brains (Ikonomidou et al., 1999). How repeated blockade of NMDA receptors during adolescence leads to PV and CB downregulation is still unclear. It has been reported that systematic injection of MK-801 for five consecutive days from PND35 to PND40 resulted in prefrontal disinhibition in rats (Thomases et al., 2013). One possibility is that downregulating PV expressions may simply serve as a compensatory response to the cortical hyperactive state, as PV deficiency could facilitate GABA release (Vreugdenhil et al., 2003), thus increasing the inhibitory signal to pyramidal neurons. Alternatively but not exclusively, the PV and CB downregulation could be ascribed to the disrupted maturation of the GABAergic signaling during adolescence. The normal periadolescent development of rat mPFC involves the upregulation of these CBP expression levels (Caballero et al., 2014). This process is activity-dependent (Philpot et al., 1997; Patz et al., 2004) and accompanies other developmental changes of cortical GABAergic neurotransmission at various levels, including expression changes of glutamate receptors, maturation of perisomatic innervation and electrophysiological properties, etc. (Le Magueresse and Monyer, 2013). Considering the essential role of NMDA receptors in regulating the periadolescent maturation of the GABAergic networks (Thomases et al., 2013), it is possible that MK-801 treatment may impede the normal upregulation of these CBPs, although the exact mechanisms awaits future studies.

We did not find significant changes of CR^+^ neuron density following adolescent MK-801 treatment. The dissociation between CR^+^ and other types of interneurons has been well documented in several post mortem studies (Reynolds et al., 2001; Zhang and Reynolds, 2002) and animal studies (Braun et al., 2007; Harte et al., 2007) and might be associated with differential distribution of glutamate receptors in these neurons (Catania et al., 1998). In addition, considering that PV^+^ and CB^+^ interneurons preferentially innervate pyramidal cells to exert inhibitory control (DeFelipe et al., 1989a,b), whereas CR^+^ neurons mainly form synaptic contacts with other types of inhibitory neurons and play a disinhibitory role (Gulyás et al., 1996; DeFelipe et al., 1999), it seems that interneurons having direct connections with pyramidal cells are more profoundly affected following MK-801 treatment.

The cingulate cortex plays an important role in cognitive control in both rodents (Ragozzino and Rozman, 2007; Kesner and Churchwell, 2011) and primates (Medalla and Barbas, 2009). In this study, we found that the mPFC interneuron deficits caused by MK-801 treatment were specific in the cingulate cortex when detected 24 h and 7 days after drug cessation. The disrupted excitatory and inhibitory balance resulting from interneuron reductions in this region may thus contribute to cognitive deficits induced by MK-801 treatment. Besides, our treatment regime did not cause obvious alterations in the prelimbic cortex, which plays a direct role in cognitive functions homologous to the dorsolateral PFC of primates (Hoover and Vertes, 2007). This does not necessarily indicate that the prelimbic cortex was spared by adolescent NMDA antagonism. Indeed, Cochran et al. (2003) found that chronic PCP treatment did not affect the number of PV-labeled cells in this region, but significantly decreased PV mRNA expression. Therefore, future studies adopting different measurements of interneuron functions are warranted to examine the potential effects of MK-801 treatment in this region.

Notably, the interneuron deficits we observed persisted 7 days after drug cessation. Consistent with a recent study showing that periadolescent exposure to MK-801 (PND 35–40) induced prefrontal disinhibition that endures through adulthood (Thomases et al., 2013), this finding indicates that NMDA receptor signaling is critically involved in the functional maturation of GABAergic circuits in the mPFC. This effect was only found in the MK-801 group of 0.2 mg/kg, which has been shown to cause long-term impairment of cognitive functions (Li et al., 2011), further highlighting 0.2 mg/kg as the appropriate dose for building animal models of schizophrenia.

In summary, our findings reveal that repeated NMDA receptors blockade during adolescence reduced the density of PV^+^ and CB^+^ interneurons in mPFC, which probably resulted from a downregulation of PV and CB expression levels, but not to neuronal death. These GABAergic deficits may be associated with impairments in mPFC-mediated cognitive functions. Taken together, our data suggest that adolescent NMDA antagonism is a useful tool for mimicking the behavioral and neuropathological changes of schizophrenia. Future studies should be conducted to elucidate the GABAergic deficits in other aspects and to evaluate putative pharmacological interventions.

## Author Contributions

X-DW and T-MS designed research; J-TL, Y-AS, H-LW and Y-YZ performed research; J-TL, Y-AS and X-ML analyzed data; J-TL, Y-AS, X-DW and T-MS wrote the manuscript.

## Funding

This work was supported by the National Natural Science Foundation of China (grant numbers 81171284, 81301152 and 81401129) and the Research Fund for the Doctoral Program of Higher Education of China (grant numbers 20120001110046 and 20130001120118).

## Conflict of Interest Statement

The authors declare that the research was conducted in the absence of any commercial or financial relationships that could be construed as a potential conflict of interest.
